# The Needs for Quality Urban Rail Transit Life in Asian Metropolitan Cities

**DOI:** 10.1007/s11482-014-9345-z

**Published:** 2014-07-14

**Authors:** Tianjiao Zhao, Kin Wai Michael Siu

**Affiliations:** The Hong Kong Polytechnic University, Hunghom, Kowloon Hong Kong

**Keywords:** Everyday life, Design needs, Quality of life, Urban rail transit, Userfitness

## Abstract

The quality of people’s lives in Asia has improved markedly since 2nd World War, with developing economies allowing people to pursue multifaceted lives instead of just working to meet basic physical requirements. In the last 30 to 40 years, urbanization in Asia has led to the emergence of urban rail transit (URT) systems in many cities. URT has become a significant part of city life, and the quality of a city’s URT experience has become a significant social issue. This study aims to comprehensively define a quality URT experience in Asian metropolitan cities using a three-pronged comparison of the URTs in three densely populated Asian cities. Both quantitative and qualitative research methods are applied in each of the three aspects studied. The research methods include a case study, a questionnaire, observation, and interviews. The different requirements of diverse groups of users are categorized and a general needs pyramid of a quality URT experience is developed. The multidimensional comparison provides useful references for designers by demonstrating the distinct attitudes of different groups of people. Our understanding of a quality subway experience is based on a needs perspective and focuses on a specific type of public space.

## Introduction

Urban rail transit (URT) is a type of high-capacity public transport found in cities, agglomerations, and metropolitan areas (Chicago Transit Authority 1974). A URT system can transport large numbers of people at high speeds with little use of land. It plays a significant role in urban management and in people’s everyday lives. The first URT, London’s Metropolitan Railway, was constructed in 1863. In the past half century, 187 URTs have been constructed around the world, and the number is still increasing (Metrobits 2013). Fifty-two of these new metros have been built in Asian cities. Lewis (2012, 3) points out that Chinese cities consider URTs as symbols of urban development (Lewis 2012). URT systems were first developed in Western countries and the requirements of Asian people are different due to their cultural backgrounds, living habits, and moral standards. In addition, as the number of URTs has increased, URTs have changed from being simple traffic tools into urban public spaces (Brooks 1997).

URT systems change people’s travel modes and their lifestyles. The URT experience has become part of everyday life and the URT space is everyday space, thus the URT experience has become a significant social space in modern society. In recent years, many mature technologies have been applied to new URT systems. Speed and accessibility are not the only features that users require from URT systems. Residents require not only an efficient URT experience, but a quality URT experience. How to design a quality URT experience is a new, multi-disciplinary research topic.

To obtain a comprehensive understanding of a quality URT experience, this study compared three dimensions of three typical Asian URTs (in Hong Kong, Shenzhen, and Tokyo). The aspects compared were the location, age, and cultural background of users. People with different backgrounds and ages were interviewed and completed questionnaires. The URTs in the three cities were used as case studies and were subjected to intensive observations. The goal of this three-pronged comparison was to gain insights into how users define a quality URT experience and how different factors affect the quality of the URT experience. The research period was 1 year and was conducted by a group of researchers. Hundreds of URT users actively or passively participated in the research. The findings of this study provide insights for future design work and provide a platform for future research into potential design opportunities.

## Review of the Definition of Quality of Life (Space)

A quality URT experience may improve the lives of citizens and the city. However, it is not easy to define “quality.” Quality is a general standard or level that represents goodness and excellence, but the nature of quality changes with different people, times, spaces, situations, and things (Kraaij and De Wilde 2001).

In *The Good City Form*, Lynch (1984, 167) describes the kind of space that can produce high quality city life. He states that a good city space should be *vital* (the environment can maintain the normal and healthy life of the individual and the survival of the species); should have *sense* (the place can be perceived and identified and this feeling activates people’s memories that create links between space and time); should be *fit* (a close match of form and behavior that is stable, malleable, and resilient); should be *accessible* (provide easy access to a variety of goods, services, and people); and should be *well controlled* (encourage harmony in society and space). All of these features can be achieved with *justice* and *internal efficiency* (see also Siu 2003, 2005)

In the sociological and psychological literature, quality of life (QoL) has been evaluated using health status (Bowling 1997), daily activities (Katz et al 1963), disability (Townsend 1962), psychological wellbeing, happiness, morale, and life satisfaction (Andrews 1986; Andrews and Withey 1976; Larson 1978). QoL is also related to the satisfaction of peoples’ needs. Sirgy (1986) argues that the higher the need satisfaction of the majority in a society, the greater the QoL of that society is. In his hierarchy of needs theory, Maslow (1943) points out that once people’s fundamental needs are satisfied, they are free to pursue higher needs such as control, autonomy, self-realization, and pleasure, which also contribute to QoL.

Carr, in his book *Public Space* (1992), describes what people need in a public space. Satisfying these needs is a necessary step in achieving QoL. As Carr states,

It is important to examine needs, not only because they explain the use of places but also because use is important to success. Places that do not meet people’s needs or that serve no important functions for people will be underused and unsuccessful. (Carr 1992, 91)

The five things that people need in public spaces are comfort, relaxation, passive engagement with the environment, active engagement with the environment, and discovery (Carr 1992). Comfort is a basic need. As with Maslow’s “physical needs” and Lynch’s “vitality” (1984), the need for comfort is satisfied by food, drink, shelter, and a place to rest when tired. It is a basic requirement of a public space, as it is difficult to perceive how other needs can be met if comfort is not obtained. A comfortable public space should provide enough sunshine, sufficient seating, and toilets. Relaxation is a higher level need and a sense of psychological comfort may be a prerequisite of relaxation. Passive engagement is another need in a public space that can also lead to relaxation. Whyte (1980) and his associate indicate that people-watching is the most popular activity in downtown plazas. People’s activities attract others’ attention. In addition to everyday human behavior, performers, formal activities, and public art all attract people to public spaces. This type of passive engagement makes public life interesting and alive. Active engagement is a more direct connection with a place and the people within it. People desire more direct contact with others and a public place can provide a link between people, and can encourage strangers to talk to each other. This social connection corresponds to Maslow’s social needs. A quality public space may lead to greater public life. As Carr (1992) states, Quality Public space (life) offers relief from the stress of work and provides opportunities for relaxation, entertainment, and social contact.

Although, QoL is studied by scholars in many different fields, there is no systematic definition or standard for assessing the quality of a URT experience. The URT experience takes place in a complex public space that is full of people with diverse cultural backgrounds, moral values, education levels, economic pursuits, and classes. As an important part of a city structure, a URT space should fulfill the requirements of a high quality public space; it should ensure that people’s URT experiences are harmonious and pleasant.

## Research Method

### Three Dimensional Comparisons

**Fig. 1:**
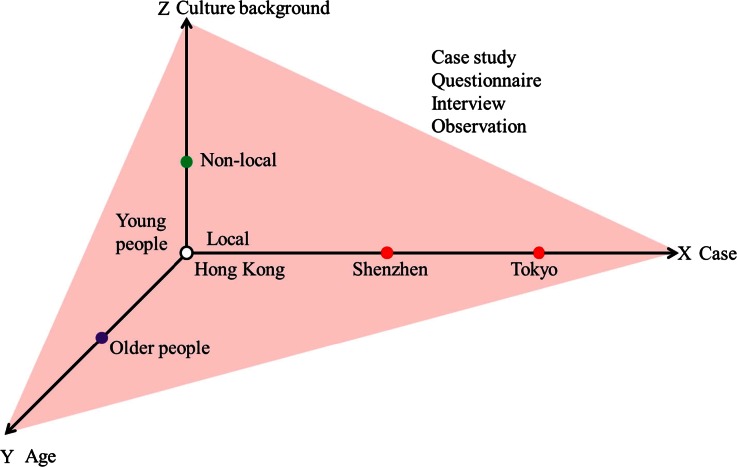
Three dimensional comparisons

In this study, both qualitative research and quantitative research methods were applied in a three-dimensional comparison (demonstrated in Fig. 1). In the X dimension, the three cities were selected as case studies. Interviews and observations were conducted to see how people perceived the URT space and used the environment on a daily basis. In the Y dimension, a questionnaire was distributed to both older users and younger users of Hong Kong’s URT in order to discover how age influences people’s requirements for quality URT life. In the Z dimension, local and non-local people were interviewed by the researchers to compare the impressions of the two groups.

This study tries to obtain valid and reliable data through direct communication with end product users. This three-dimensional research provides a comprehensive research method that covers the most important aspects of assessing URT life. After comparing the three basic aspects, the researchers organized the findings to discover the regulations behind these phenomena.

### Case Study

Considering the objectives and practical constraints of this study, an exploratory case study approach was adopted. The case study approach has many advantages in urban research, particularly for understanding and interpreting observations of social phenomena (Merriam 1988). In this study, the case study contains two stages. The first stage was to discover and describe the case of the three urban rail transit systems of Hong Kong, Shenzhen, and Tokyo. The second stage was to focus on Hong Kong’s Mass Transit Railway (MTR) to achieve an in-depth exploration of the key stations.

The Tokyo Metro typifies the meaning of metro in a global context. As the first URT in Asia, it has an 86-year history. The development of Tokyo’s society and economy has caused the Tokyo Metro to have a complicated history of development. The abundant use of the Tokyo Metro and the historical element of the metro’s story endow the Tokyo Metro with a unique but representative character.

Hong Kong’s mass transit railway (MTR) is selected as a core case. It is famous worldwide for its security, stability, and high quality of service. It has served as a model for many other Asian cities’ URT projects. It carries an average of four million passengers each day (MTR n.d.) and has become the most popular transport tool in Hong Kong. Hong Kong is an international city with people of diverse cultural backgrounds, so it is very important that the MTR functions appropriately with diverse expectations and behaviors (Cowell 2012). For the purpose of this study, the East Rail line was chosen as the secondary case, as it covers the main shopping area, residential area, and working area of Hong Kong (Census 2011).

Shenzhen Metro was constructed in 1999, which is very recent compared to Tokyo and Hong Kong’s UTRs. As a Special Zone, Shenzhen, China, is filled with young people from different provinces. It is a lively, multicultural city. The difference in social environment and era of production has created a modern metro that has benefited from the experiences of the other two metros and developed a unique style.

The research results from these cities are comprehensive in the context of Asian UTR studies, as the URTs selected accommodate different cultural and demographic aspects in Asia, were constructed in different times, and developed at diverse prosperity levels.

### Observation

The observations were recorded with a camera and qualitative descriptions. Observation was conducted over time and in various locations. The geographical research area encompassed the whole station, including the pass way platform and train compartment. The observation was conducted from Monday to Friday, during weekends, and on special days and holidays.

### Questionnaire

Questionnaires were distributed to both the young people and older people in Hong Kong. Young people who were living, studying and working in Hong Kong were asked to complete the questionnaire using the internet, as internet access and computer ability are quite common for this demographic (Castells 1996/2001). For older URT users, the questionnaires were conducted on paper, in a community center that is primarily used by the elderly. Ma On Shan Older People Community Center was selected by the researchers for this purpose because the center is located near the Ma On Shan station, so is likely to be frequented by older people with abundant MTR travelling experiences.

### Interview

An interview is a direct way to obtain users’ opinions. The one used in this study is both effective and valid (Merriam 1988). Users were interviewed in the three cities. Participants were assigned to different groups, including residents, new residents, foreign residents, and visitors. The interviews were conducted with young people and older people. The in-depth interview with these URT users provided insight into different users’ expectations of and requirements for the URT. The interview included questions like, “How do you define a quality URT life?”, “Are you satisfied with current URT using experiences?”, “What points are you not satisfied with? What points are you most satisfied with?”, and so on. Some extemporaneous questions were also asked during specific communication. The conversations were audio recorded.

## X Dimension—The Quality of the URT Experience in Different Cities

### Features of the URT Systems in the Case Cities

The observations of the three case cities revealed both similarities and differences in the three URTs. All of the city metros possess more or less the same design, but each has its own unique characteristics. When discussing a particular URT system, people often used similar representative words to describe it. These positive and negative words reflected the characteristics of the URT system in each city. These characteristics have gradually developed to become features of the URT experience.

**Fig. 2:**
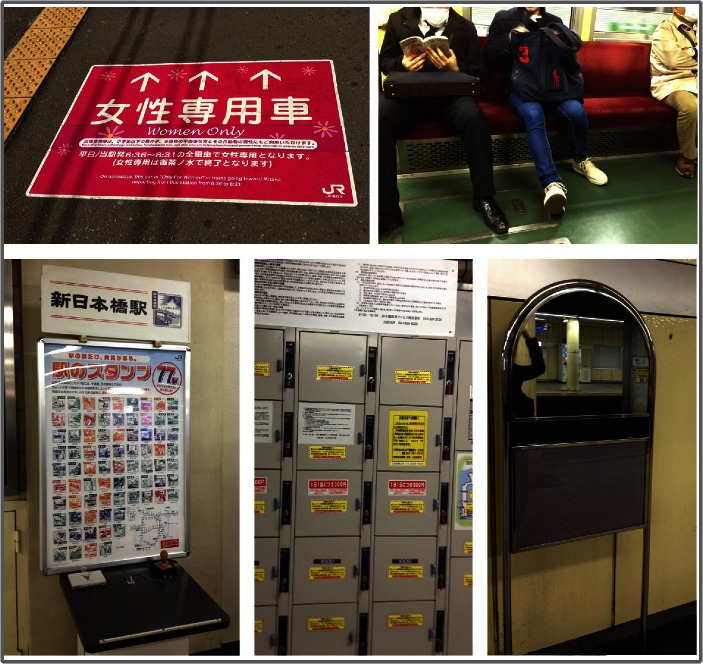
Design details in the Tokyo Metro

The Tokyo Metro is an excellent example of how a URT can accommodate human users. It has many special features, including storage cabinets, women-only compartments, public mirrors, metro medals, heated seats, and so on (as shown in Fig. 2). These are just some of the features that contribute to the high quality of the human services in Tokyo’s URT. Although the environment and the facilities have been used for a long time and look old, the human services always create a positive experience for the users. People can easily find the solutions to their specific needs in this environment. This detailed design makes people feel safe, comfortable, and relaxed. Other obvious characteristics of the Tokyo Metro are the crowding, fast speed, and quiet environment. These are not features of the facilities, but of the people who use the Metro. The environment is alive and full of energy. Users’ behavior is thus a significant element affecting the character and quality of the space.

**Fig. 3:**
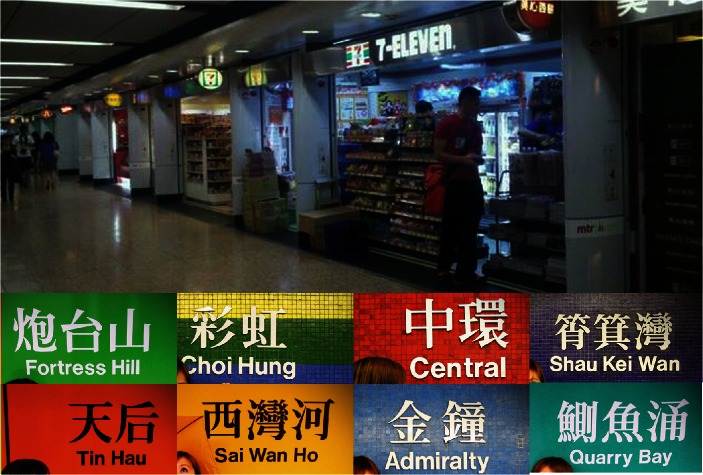
Metro shops and colorful decoration in Hong Kong’s MTR

The aesthetic appearance of Hong Kong’s MTR is one of its most distinguishing characteristics (Fig. 3). The colorful mosaic wall has become a symbol of Hong Kong. In addition, the various metro shops demonstrate the strong commercial atmosphere inside the MTR, which is a great convenience for users. The MTR has existed for 30 years. Since the 1990s, the number of MTR shops has increased. In recent years, MTR shops have been supplying people with their daily requirements including clothes, food, banking, barber shops, etc. Some stations even have rows of storefronts. This is the distinguishing feature of the Hong Kong MTR that is not evident in the URTs of the other two cities. The different kinds of MTR shops also demonstrate the significance of the MTR experience and the multiple functions of the MTR space in Hong Kong.

**Fig. 4:**
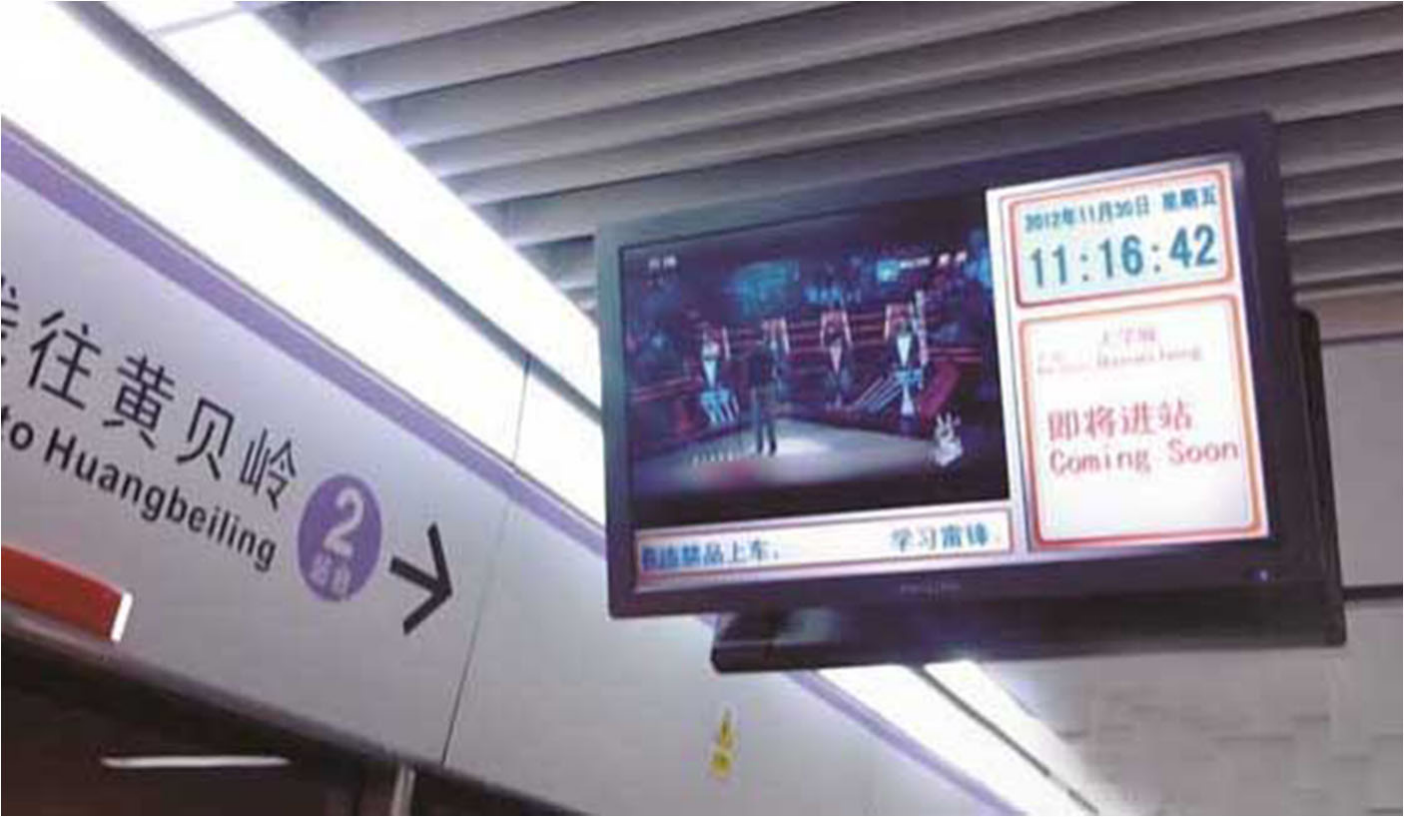
TV program in the Shenzhen Metro

The Shenzhen Metro is a newly constructed metro and its characteristics are not as obvious as the other two URTs. Although everything is newly constructed and the basic facilities are well arranged, the facilities are not human enough and the service quality needs to be increased. The image of the Shenzhen metro is not as impressive as that of the Tokyo or Hong Kong metros. The environment is not distinctive or impressive, and it does not have many stores. It has a short history and distinctive characteristics generally develop over time. However, Shenzhen metro is vigorous due to its users. As a city full of young people, Shenzhen has a metro that is also full of energy. There are few older people in the Shenzhen metro. The TV shows and advertisements broadcast in the stations appeal to the city’s young demographic. Figure 4 shows the Shenzhen Metro TV tuned to a famous TV show, *The Voice of China*, which is quite different from the political news and MTR rules that are shown on Hong Kong MTR TVs. Many users in the Shenzhen Metro stop to watch the TVs.

These are just the obvious characteristics of the URTs in these three cities. In this study, we also interviewed city residents and asked them to describe the URT experience in their city. These descriptions are summarized in Table 1. The users’ descriptions provide real evaluations of the URT experience. Although each metro has its own special characteristics, all three satisfy people’s basic needs, which are more or less the same across all cities and demographic groups.

**Table 1:** Description of each city subway

Subway image	
Tokyo metro	Silent, human, historical, fast rhythm, accessible, detailed, can buy food, enough toilets, old, indifferent, deep, convenient.
Hong Kong metro	Clean, low temperature, safe, convenient, frequent, punctual, chaotic, orderly, flourishing, crowded, high priced, fast, human, civilized, global, multiple elements, callous, MTR shops, unacquainted, flexible, artistic.
Shenzhen metro	New, reasonable price, crowded, chaotic, fancy, emotional, clean, comfortable waiting environment, airtight, modern, alive, novelty, unclear information, spacious.

### Common Factors Affecting the Quality of a URT Experience

To obtain comparable data for the different cities, users’ URT journeys were divided into several processes for analysis. In the interviews, the users described the features that influenced the quality of their daily URT experience.

The URT space is a web that people enter and exit every day. Unlike other public spaces, the URT is not a point, but a web that separates the passengers from the city. People’s URT experience occurs in URT space and can be divided into seven stages: entering the MTR, walking to the platform, waiting on the platform, in the compartment, transferring trains (if needed), walking to the exit, and leaving the station. In the interviews, the interviewees were encouraged to speak their minds on everything that influenced their URT experience. All of the factors discussed in the interviews are summarized in Table 2.

**Table 2:** Influences that affect the quality of people’s URT experience

A: Before entering the subway; B: At the entrance to the compartment; C: Inside the compartment; D: From the compartment to the exit; E: Exiting the subway station	
A	Distance between home and station. Ease of finding the MTR entrance in the city.
B	Facility barriers. Cleanliness of environment. Convenience of payment method. Price. Clarity of instructions. Punctuality of trains.
C	Safety and reliability of trains. Availability of seating. Availability of Wi-Fi. Availability of free newspapers. Provision of interesting TV programming.
D	Transfer distance. Availability of toilets. Convenience of shops. Provision of interesting advertisements. Provision of attractive artwork.
E	Efficiency of feeder bus service.

### People’s Attitude Toward the Quality of Their URT Experience

Although the 20 factors identified occurred in all three cities, the amount of enthusiasm and dissatisfaction varied between cities.

Most Hong Kong residents were satisfied with the basic functions of the MTR; they ere dissatisfied with insufficient toilets and the noisy, indifferent environment. In Shenzhen, some residents were unsatisfied with some of the basic functions of the UTR. Most of these dissatisfied users had experienced the Hong Kong MTR and the Shenzhen Metro, and they compared the instruction system of the Shenzhen Metro unfavorably with that of the MTR. Tokyo residents were satisfied with the basic functioning, details, and accessibility of the Metro, but highlighted significant problems with crowds, particularly during rush hour. This was also a common complaint in Hong Kong.

The three metros represent different levels of development, and people’s concerns were centered around different in the three systems. Some of these concerns are caused by poor design, some are caused by people’s behavior, and some are only noticeable because of comparisons with other systems. Each city metro has its own characteristics, but the people in all three cities have the same basic requirements for city metro development.

The observations and interviews gave a comprehensive picture of the URT experience in the different cities, including their unique features, similar requirements, and the different attitudes of their users. There were similarities and differences among the URT users in the three different cities. This same pattern was seen among people of different ages.

## Y Dimension—The Quality of the URT Experience for People of Different Ages

### The Results of the Questionnaire

The researchers used the factors identified in the above interviews to design a questionnaire for Hong Kong MTR users. The participants were asked to evaluate the importance of each factor on a scale ranging from 1 (less important) to 5 (quite important) (Li 2007). As many older people find Wi-Fi difficult to manage or understand, it was not included in the older people’s questionnaire. The questionnaire sample included 60 completed questionnaires from young people and 25 completed questionnaires from older people. The ranking of each factor is shown in Table 3.

**Table 3:** Ranking of the factors affecting the quality of the URT experience by age group

Ranking		Influences of URT life quality	Mean	
Young	Old		Young	Old
1	5	Safe and reliable trains	4.75	4.31
2	7	Clear instruction facilities	4.53	4.19
3	6	Punctual trains	4.52	4.27
4	12	Reasonable price	4.42	3.52
5	9	Clean station and compartment	4.37	4.095
6	8	Easy to find the MTR entrance	4.35	4.12
7	4	Short transfer distance	4.03	4.35
8	1	Enough toilets	4.02	4.50
9	–	Available Wi-Fi	3.98	–
10	2	Barrier-free facilities	3.93	4.48
11	13	Convenient payment method	3.93	3.42
12	11	Short distance between home and station	3.93	3.62
13	10	Efficient feeder bus service	3.92	4.09
14	3	Enough seats	3.77	4.40
15	14	Convenient MTR shop	3.42	3.33
16	18	Free newspapers	3.1	2.67
17	15	Attractive artwork	2.83	2.81
18	16	Interesting TV programming	2.75	2.76
19	17	Interesting advertisements	2.62	2.71

The factors were ranked by the URT users. The questionnaire referred to a specific location, but the needs were similar in all of the URTs. Among young people, the influential factors can be summarized into five groups, from most to least important: *A*—functional needs (safe and reliable trains, clear instructions, and punctual trains); *B*—economic needs (prices); *C*—physical needs; *D*—entertainment needs (available Wi-Fi, convenient shops, and free newspapers); and *E*—art needs (attractive artwork and interesting advertisements). Functional needs are the elements related to the basic travel experience. Economic needs are related to the necessary consumption in the URT space. Physical needs are related to the requirements of the body, such as clear air for breathing, less labor to save energy, and less thinking to save time. Entertainment and art are needed to make life interesting.

### Quality of Experience for Young People

Young people are a special group of MTR users. They belong to the low-income group and most of the young people in the city are at the beginning of their careers. Modern young people have their own lifestyles and are under great social pressure, especially in a fast-paced city such as Hong Kong. During rush hour, the MTR is full of young passengers. People walk fast with no expressions on their faces. Many young business men wear the black and grey formal clothes called the Hong Kong dress. Their fast pace shows that people in Hong Kong treasure time and live at a high speed. There are a great many young people who save time by walking on the escalator instead of standing on it. When young people wait on the platform or enter a compartment, they are frequently playing on their mobile phones.

**Fig. 5:**
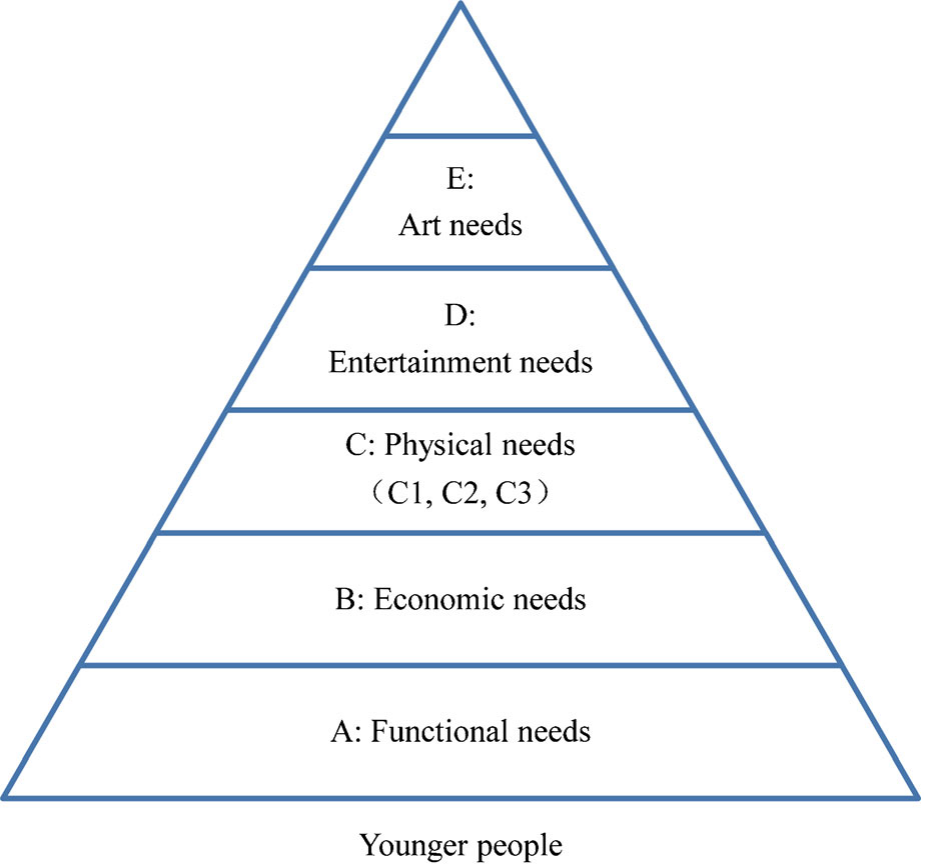
Needs pyramid for younger people

The questionnaire revealed that young people considered functional needs the most important, economic needs second, followed by physical needs and entertainment needs. This hierarchy is illustrated in Fig. 5. The most important needs—the ones that obtained the highest score-are located at the bottom of the pyramid and are the foundation of a high quality experience. The less importance factors are higher in the pyramid. For younger people, functional needs were the most important; they wanted to use the least time to finish the most things. This is also in accordance with the character of modern people. Cost was the second most important factor, due to the low income of the young people and the high pressure from their work and society. Young people do not receive any preferential treatment from the government and transportation costs are a large percentage of their daily expenses. A sustained rise in MTR prices creates great economic hardship for them. Physical needs were only the third most important factor, as they do not strongly affect young people’s work and economic situation. Young people have strong bodies, so they do not care too much whether they have a seat, whether they can find the toilet easily, or whether they need to stand for a long time. Entertainment seemed to be more important than art, which also demonstrated the “snack culture” among modern young people. Entertainment brings instant happiness, whereas art can only be appreciated when the users slow down.

#### Quality of the URT Experience for Older People

**Fig. 6:**
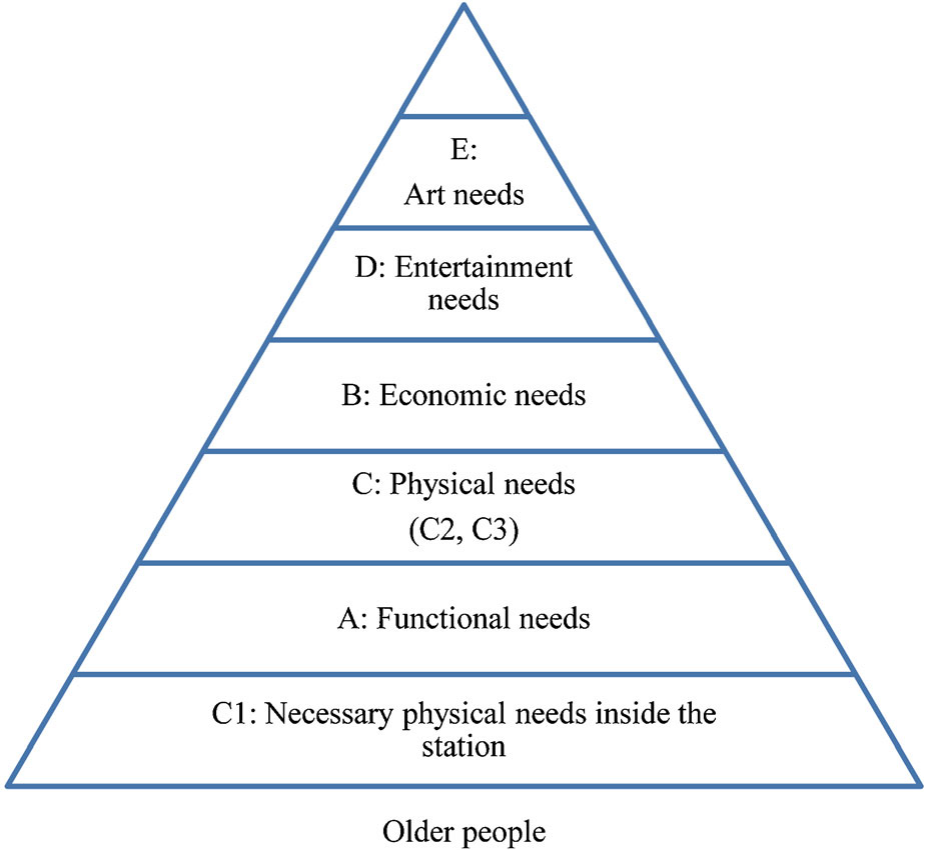
Needs pyramid for older people

The needs pyramid was a little different for older people. Older people are more concerned with physical needs than young people. Economic needs are less significant because the government provides incentives for older people (Public Transport Fare Concession Scheme for the Elderly and Eligible Persons with Disabilities) (MTR n.d.). For older people, physical needs can be subdivided into three groups: *C*_*1*_—inside environment needs (clean station and compartment); *C*_*2*_—necessary physical needs inside the station (barrier-free facilities, enough seats, and short transfer distances); and *C*_*3*—_needs related to the city (easy to find MTR entrances, short distances between home and station, and efficient feeder bus services). Entertainment needs and art needs include factors that enrich people’s lives. The pyramid for the older people is shown in Fig. 6.

The above pyramid illustrates the key factors that influence the quality of the UTR experience for older people and the features that could be improved. Older people’s requirements can be summarized as follows.

- *Physical comfort*
  Aging causes many physiological changes including changes in sight, hearing, memory, and body flexibility (Kwok 2006). These physical changes create special requirements that significantly influence the subway experience, as shown in the following quotation.
  Respondent: I don’t like to go out during rush hour. There are so many people in the station I cannot get a seat during the whole 30 min journey. This makes me very tired. But I don’t think this issue can be solved, as the MTR already provides a frequent service. There are too many people in Hong Kong now.
  A peaceful environment was important to the older users of the MTR. A rocky train can be difficult for older people and they cannot stand up for long journeys. If the problem of overcrowding cannot be solved quickly, it is important to not only ensure that there are priority seats for the elderly, but also to instruct younger people to offer these seats to them.
  In addition to the seating problem, toilet facilities are also important for older people. There are currently not enough toilets at MTR stations and when asked about toilet facilities, the interviewees unanimously replied that they were not satisfied with them.
  Respondent: Currently there are not enough toilets in the station, and they are too small. I have to walk a long way to find the toilet-this really needs to be improved.
  Among the MTR’s 84 stations, only 37 have a toilet inside the station. Ten of them are inside the payment area, and most of them are located near the entrance to the station. It is a long way for the elderly from the train compartment to the toilet.
  The respondents raised three points about this issue. First, they often did not know whether there was a toilet nearby. Second, they did not know where the toilet was located. Third, even if they did know the location, it was inconvenient for them to walk a long distance to get to it. Greater consideration should be given to this issue. Although all of the adults were aware of these issues, these problems seemed more serious for older people.
- *Independence*
  Independence is important to QoL because it enables people to get outdoors, enjoy life, meet people, and avoid having to rely on others (Gabriel and Bowling 2004). In the MTR environment, independence is also an important factor as older people find it difficult to control their direction, speed, and body in a crowded public space. Many of the older interviewees said that they did not like going out during the rush hour because they cannot walk as fast as younger people. The moving crowds pushed them to walk faster, making them feel quite uncomfortable and out of control. Older people try to control their life rhythm, as demonstrated by the sub-theme “having freedom as opposed to limitations” (Borglin et al. 2005). Efficient barrier-free facilities, feeder bus services, and a short distance between home and station were also significant factors for older people.
- *Suitable entertainment*
  The results of the observations revealed that there was insufficient entertainment for older people in the subway. The questionnaire results suggested that the respondents did not consider this to be an important feature, yet they also rated it as unsatisfactory.
  Interviewer: Do you feel bored in the compartment?
  Respondent: I don’t care about it because I think there is no way to solve this. The TV program is discontinued when they announce the station name. We cannot hear the TV in the noisy environment. In the crowded compartment it is even difficult for us to get a seat so how we can read the newspaper? I choose to listen to the radio sometimes, like on the bus. But unlike the bus, the train is quite fast and the time between each station is quite short. Even if I want to listen to music, I have to maintain vigilance to see whether I should get off the train.
  According to Gabriel and Bowling (2004), social entertainment is one of the most significant factors affecting the QoL of older people. Social entertainment makes older people feel busy and provides mental stimulation. Although people pay less attention to entertainment in the subway space, this aspect should not be ignored. Older people are not interested in current trends such as mobile phones, which entertain younger people. Some suitable types of entertainment should be designed for older people to improve the quality of their subway journeys.

## Z Dimension—The Quality of the URT Experience for Locals and Non-Locals

Interviews with five local Hong Kong residents and three non-local residents showed that they had different ideas about URTs. Their answers showed the different attitudes of people with different cultural backgrounds. The interviews were based on three main questions: 1) how do you feel about the MTR space?; 2) how would you describe a quality URT experience?; and 3) what are the advantages and disadvantages of the MTR, in your eyes? Some other questions were asked randomly during the conversations.

The local people had great memories of the MTR. Their descriptions exhibited a sense of responsibility and emotion. For local residents, the MTR has been around for a long time. Both the positive and the negative attitudes were expressed with abundant emotion.

Local interviewee: I took the MTR to school every day when I was young and now it takes me to work. I have a lot of memories about going to school by MTR with my friends. The MTR has expanded over what it was before. When I take the Ma On Shan Line, which I took every day when I was young, I feel amazed and warm when I see the old decorations! It seems to bring me back to childhood! I suddenly feel I am old! Ha ha ha. (Local Hong Kong resident)

Local interviewee: I feel the MTR has changed a lot. Several years ago, they didn’t distribute the newspaper, we didn’t use the Octopus, and it was not as accessible as now. Every great advance in the MTR made me happy. I even feel a little proud about the MTR. Now when I see a colorful mosaic, I feel it is a logo of the Hong Kong MTR. Although this design does not reflect the Hong Kong culture at the beginning, but it has become part of Hong Kong culture now. A quality URT life, er… I think it should be quiet and comfortable, with civilized people. (Local Hong Kong resident)

Local young people were both critical and proud of the MTR. They had complex expectations of the MTR experience. Local people were more sensitive to details than the non-locals. They connected their basic transportation needs with their everyday lives, political viewpoints, and old memories. By consulting with local young adults, designers may identify specific design opportunities.

Local interviewee: A quality URT experience in my eye should be user-centered in every aspect. I feel unsatisfied about the price of the MTR. The MTR Company has already earned a lot from the real estate and they are still greedy. They constructed the MTR in an open space and then constructed real estate on the open space to earn money. The poor bottom dwellers have to walk a long way to the station. For the basic function, I feel it is OK. It has been improved year by year. By the way, I hope the MTR will consider bicycles users of the MTR in the future. Besides, I feel the MTR is too noisy. I prefer the environment of the Tokyo URT. (Local Hong Kong resident)

The attitudes of the non-locals toward the MTR were calmer and simpler. They focused on the basic functions rather than on higher-level requirements, and did not have the same emotional investment in the city. The longer young people stayed in the city, however, the more harshly they criticized the URT.

Non-local interviewee: I think the Hong Kong MTR is good enough compared to the subway in China’s mainland. It is humanized and well designed. I do not expect some higher service from the MTR, such as MTR shops or artistic work. To improve the functionality is more important than other things. I can just view this city in a bystander’s way, as I don’t think it is easy for me to integrate into this society. So I don’t expect too much. (Interviewee from China mainland who was studying in Hong Kong)

Non-local interviewee: I think a quality MTR should be clean, safe, and well-managed. The Hong Kong MTR is good enough. I don’t have trouble reading the information, as the whole city is so international. (Foreigner working in Hong Kong)

The non-local interviewees did not have emotional responses to the environment and did not obtain emotion rewards from the environment. Some of the non-locals considered this city as a temporary working place, rather than a place where their future family will live.

Non-local interviewee: I feel the MTR environment is ice cold. Of course the air conditioning is low. But I do not mean that. I do not feel emotional care in the MTR. People stay in their own worlds, walk fast, and speak fast. Advertisements change as frequently as the passengers in the MTR. In this strange environment, actually, I feel alone. (Person from the Chinese mainland who was working in Hong Kong)

The interviews revealed young people’s feelings about the MTR. People’s requirements for a positive experience differed according to their culture backgrounds and their emotional connection to the city. The longer they stayed in the city, the more harshly they criticized the URT. Local people focused on high-level needs, whereas non-locals focused on low-level needs. Although these groups identified different levels of needs as important, their URT needs more or less followed the same hierarchical order.

## Quality URT Experience Pyramid

**Fig. 7:**
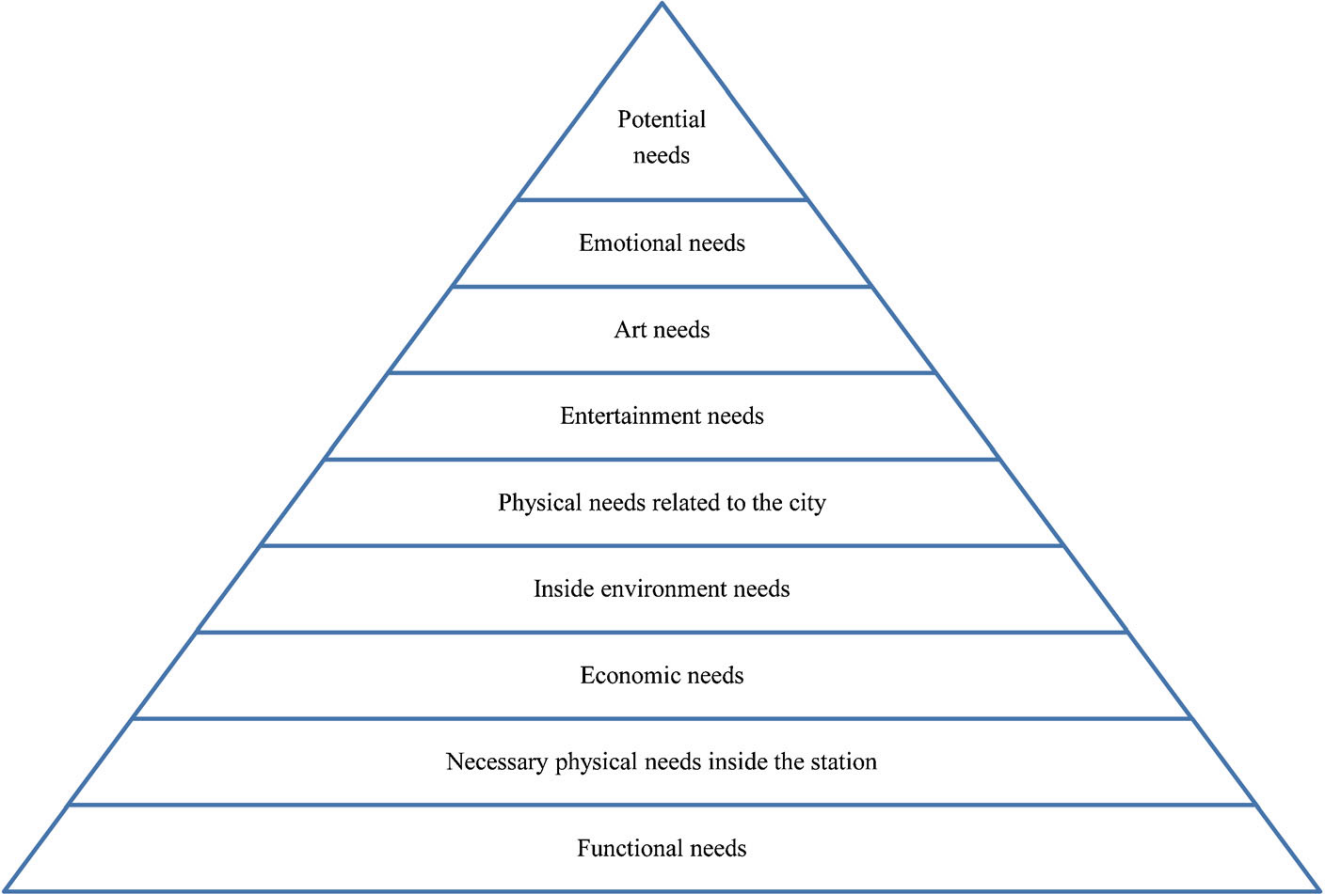
Quality URT experience needs pyramid

The diverse groups of users discussed above revealed a mixture of different and similar URT needs. As the URT is shared by people of different backgrounds with different needs, the researchers identified the general requirements that best fit everyone’s needs. A general pyramid was constructed by combining the young and old peoples’ pyramids. In addition to the elements used in the previous pyramids, both the local and non-local interviewees referred to emotional needs in their interviews (through the use of such words as “alone” and “memories”). The emotional needs were felt by users, but are difficult to measure. These less obvious needs were placed at a high level of the combined pyramid, just below “potential needs.” The integrated URT-needs pyramid shown in Fig. 7 illustrates an overall quality URT experience.

In this general needs pyramid, the most significant needs are located at the bottom, whereas the less necessary elements are located higher on the pyramid. This needs pyramid outlines a quality URT experience for all users. Just as with Maslow’s needs theory, the lower level needs in this pyramid should be fulfilled first to ensure a quality URT experience. Furthermore, the width and height of the pyramid will affect the quality. The low level needs determine the stability of the pyramid, whereas the high level needs affect the more subtle aspects of the experience’s attractiveness to users. If each area of the pyramid is considered a step in the quality of the experience, a stable, high quality construction corresponds to a high quality URT experience—one with a strong, wide base that supports many high level attractions. Over time, more detailed potential needs can be added to the pyramid.

This outline of URT needs was based on users’ perspectives and organized by professional researchers. It is a general needs pyramid. The low level needs play the most significant roles in people’s URT experience and are easy to fulfill and perceive. Higher on the pyramid, needs become more difficult to identify and fulfill. However, as with Maslow’s (1943) needs, the pyramid does not describe an absolute hierarchy. Different people expressed different patterns in the hierarchy of needs and different groups gave greater priority to different needs. However, the final pyramid follows the general trends indicated by all of the participants.

When low level needs are fulfilled, people begin to focus on their high level needs, and these higher level needs will alter users’ behavior for both objective and subjective reasons. The objective perspective focuses on the external conditions contributing to QoL, whereas the subjective perspective is based on people’s internal judgment as it relates to QoL (Pichardo-Muñiz 2011). For instance, people focus on their high level needs in a more developed URT space. People in Shenzhen noticed the lack of instructions in the Shenzhen Metro, whereas people in Hong Kong focused on other details. Personal differences also affect users’ perceptions. In the same environment, local people focused on their high level needs, whereas the non-locals only cared about the basic needs. When designing a quality URT, designers should consider what needs users’ are focused on and should consider design opportunities that suit the characteristics of various user demographics.

## Conclusions

Many studies have attempted to define QoL and to measure it from an analytical perspective. Some authors have suggested that QoL studies are most suited to the study of urban life, due to the contradictions characterizing urban environments (Pichardo-Muñiz 2011). The urban railway transit space is a common public space shared by different users, and thus is a suitable place to conduct QoL research. A quality URT experience can improve city dwellers’ lives, as it is a significant part of the city system.

This study focused on specific public areas in Asia. The three-pronged comparison guaranteed the reliability and comprehensiveness of the findings. The opinions of different people in the same environment, of different people in different environments, and of the same people in different environments were all considered.

The results not only demonstrate the differences in each comparison dimension, but also reveal the similarities in people’s requirements. Both the differences and similarities can be used to inform future design work. The quality URT-needs pyramid constructed in this study connects abstract requirements to specific design features. This study provides a creative approach for defining a high quality URT experience based on the needs perspective. The findings are expected to have long-term effects on design and policy making. Although the selected informants are representative (i.e., young people, older people), further studies should expand the sample size and the number of locations to increase our understanding of users’ needs. Future studies should also focus on particular groups of informants so that a more in-depth understanding can be generated.
